# Pruning the Communication Bandwidth between Reinforcement Learning Agents through Causal Inference: An Innovative Approach to Designing a Smart Grid Power System

**DOI:** 10.3390/s22207785

**Published:** 2022-10-13

**Authors:** Xianjie Zhang, Yu Liu, Wenjun Li, Chen Gong

**Affiliations:** 1 Key Laboratory for Ubiquitous Network and Service Software of Liaoning Province, School of Software, Dalian University of Technology, Dalian 116620, China; 2 School of Computing and Information Systems, Singapore Management University, 81 Victoria Street, Singapore 188065, Singapore; 3 Institute of Automation, Chinese Academy of Sciences, Beijing 100190, China

**Keywords:** smart grid, deep reinforcement learning, cooperative agents, communication, causal model, estimating ITE, variational auto-encoder

## Abstract

Electricity demands are increasing significantly and the traditional power grid system is facing huge challenges. As the desired next-generation power grid system, smart grid can provide secure and reliable power generation, and consumption, and can also realize the system’s coordinated and intelligent power distribution. Coordinating grid power distribution usually requires mutual communication between power distributors to accomplish coordination. However, the power network is complex, the network nodes are far apart, and the communication bandwidth is often expensive. Therefore, how to reduce the communication bandwidth in the cooperative power distribution process task is crucially important. One way to tackle this problem is to build mechanisms to selectively send out communications, which allow distributors to send information at certain moments and key states. The distributors in the power grid are modeled as reinforcement learning agents, and the communication bandwidth in the power grid can be reduced by optimizing the communication frequency between agents. Therefore, in this paper, we propose a model for deciding whether to communicate based on the causal inference method, Causal Inference Communication Model (CICM). CICM regards whether to communicate as a binary intervention variable, and determines which intervention is more effective by estimating the individual treatment effect (ITE). It offers the optimal communication strategy about whether to send information while ensuring task completion. This method effectively reduces the communication frequency between grid distributors, and at the same time maximizes the power distribution effect. In addition, we test the method in StarCraft II and 3D environment habitation experiments, which fully proves the effectiveness of the method.

## 1. Introduction

Context, Literature and Limitations: With the gradual increase in electricity demand, the power grid system meets the needs of a more diversified market while ensuring safe, reliable and stable performance, which brings great challenges to the traditional power grid [1,2,3]. Therefore, the next-generation power grid system, the smart grid, is designed to meet the above challenges [4]. It is based on a more intelligent power distribution method to ensure the stability of users’ electricity consumption [5], and requires the simultaneous cooperation of multiple distributors on the network to meet the customers’ needs [6]. To achieve this, researcher begin to use multi-agent reinforcement learning algorithms to solve collaborative decision-making in the smart grid power systems [7,8,9,10]. In this framework, the distribution nodes in smart grid are modeled as the reinforcement learning agents to learn cooperative distribution strategy [11].

Several collaborative reinforcement learning algorithms have been proposed recently to address the collaborative problem in power grids [12,13,14,15]. These algorithms model nodes on the smart grid, such as power distributors, charge and discharge controllers, and switches controllers, as agents, and improve the performance of the overall system by optimizing the cooperative strategy of multiple nodes (agents). A distributed reinforcement learning algorithm based on the representation of cross node distributed value function is proposed, it is demonstrated in the simulation of distributed control of power grid and has achieved good results [12]. A multi-agent system (MAS) for managing distribution networks connected to regional substations is proposed. Using MAS, manage the orderly connection and disconnection of resources to minimize disruption to the balance of supply and demand within the grid network [13]. A multi-agent algorithm formulated within the framework of dynamic programming to solve the controlling power flow problem proposed, where a group of individual agents can autonomously learn to cooperate and communicate through a global optimization objective [14]. A fuzzy Q-Learning approach is proposed to treat microgrid components as independent learners while sharing state variables to coordinate their behavior. Experiments have proved the effectiveness of a single agent to control system components, and coordination can also systematically ensure a stable power supply and improve the reliability of microgrid systems [15]. In these methods, there must be sufficient information exchange between agents, including but not limited to each agent’s local observation state, and accepted reward, so that the coordination of the agent system can be realized. However, in the actual smart grid system, the number of the nodes (agents) is large, resulting in a complex grid network, which requires a considerable communication bandwidth to meet the communication needs [16]. As a result, there has been renewed interest in the academic community to reduce the communication bandwidth between agents [17].

In reality, information exchange through communication enables humans to form a team, allowing everyone to perceive, identify, and coordinate actions in the physical world to complete complex tasks [18]. This mechanism also applies to systems where multiple intelligent agents need to cooperate [19]. In a multi-agent system, agents only have local observations, and communication allows them to share information and spread experience. In embodied agents [19,20,21], for navigation tasks, the navigator agent needs additional communication from the “oracle agent”, which provides detailed information of the map. For the study of communication among agents, many algorithms have been proposed recently, including DIAL [22], CommNet [23], BiCNet [24], TarMAC [25], NDQ [26], and CoMON [19]. However, these methods require continuous communication among agents. As the number of agents and the communication frequency increase, the valuable information may be submerged in the ocean of information. In some practical applications, communication is expensive, and the increase in the amount of information causes an increase in bandwidth, computational complexity, and communication delays.

To allow the agents to identify valuable knowledge in the ocean of information, some studies applied the attention mechanism to learn the weight for the communication among agents to filter redundant information [25,27]. However, the time-series observation data makes the learned attention model unstable [28]. Besides, the attention model does not essentially reduce the communication bandwidth among agents but only imposes a weight on the information [9]. There is another option to reduce communication bandwidth, which is to use the selective sending mechanism. The key to designing a selective sending mechanism is managing to make the agent identify the critical state and moment to send a message. If we can predict the outcome of sending and not sending a message, then the agent can selectively send messages based on the predicted outcome. Fortunately, in causal inference, whether or not to send a message can be viewed as an intervention variable (binary variable), and the effectiveness of the intervention variable on the outcome can be predicted by estimating individual treatment effect (ITE) [29,30,31]. Therefore, we propose a causal inference communication model to address the communication bandwidth problems among agents. By employing causal inference, we regard whether to or not communicate as a intervention variable. Based on the intervention variable, we can estimate the effect of communication on the outcome to determine the intervention variable and decide whether the agents need to communicate or not. The learned causal model allows agents to send messages selectively, which reduces the amount of communication exchanged between agents, thus reducing the communication bandwidth.

This paper proposes a collaborative multi-agent model with causal inference, referred as the Causal Inference Communication Model (CICM), to help each reinforcement learning agent in the system decide whether to communicate or not. Specifically, we implement CICM using the following steps. First, to connect reinforcement learning with causal model, CICM embeds the control problem into a Graphical model with Bayesian formula, which is a causal model at the same time. Then, we parameterize the causal graph as a latent variable model based on Variational Autoencoders (VAE) [32]. By evaluating the ITE [30], we can determine whether the communication is beneficial. Thus, agents can only communicate at critical moments and necessary states, reducing the communication bandwidth in the smart grid system.

Therefore, a summary of our objectives is listed below:

(1) To apply the causal model to optimize the communication selection in the problem of the smart grid, also in the collaborative agent tasks.
(2) To formulate a graphical model which connects the control problem and causal inference that decides the necessity of communication.
(3) To prove empirically that the proposed CICM model effectively reduces communication bandwidth in smart grid networks and collaborative agent tasks while improving task completion.

Contributions of this Paper: This paper makes the following original contributions to the literature in these following areas:

(1) This is the first study to apply the causal model to optimize the communication selection in the problem of the collaborative agent tasks in smart grid.
(2) A new graphical model is developed which connects the control problem and causal inference that decides the necessity of communication.
(3) Innovative numerical proof that the proposed CICM model effectively reduces communication bandwidth is provided.

Structure of the Paper: Section 2 presents the existing literature on the smart grid, communications in cooperative agents, and causal inference and their limitations. Section 3 discusses the concepts and structure of a graph model to adopt in this study, while Section 4 presents the causal graphical model and the inference method, Causal Inference Communication Model (CICM). Data sets, the environment and computational experiments are reported in Section 5 and the results of these experiments are analyzed in Section 6. Research limitations and threat to validity are discussed in Section 7. Conclusion and further research are stated in Section 8.

## 2. Related Works: Smart Grids, Communications in Cooperative Agents, Causal Inference

This section discusses related works, including the smart grid, communications in cooperative agents, and causal inference. Firstly, the recent research progress of smart grid distributors is introduced. It is a trend to model the distributors in power system as reinforcement learning agents. Because of the problem of communication bandwidth between power grid systems, the research progress of multi-agent communication research is further discussed. Finally, causal inference algorithms for reinforcement learning are introduced.

### 2.1. Smart Grid

Smart grids are functionally able to provide new abilities such as self-healing [33], energy management [6], and real-time pricing [5]. The realization of the above functions requires advanced technologies such as intelligent power distribution, distributed power generation, and distributed storage [34]. Of which, intelligent power distribution plays a key role in the smart grid, which is the intermediate component connecting power generation and consumers [35]. Much work has been done on the optimization of power distribution systems in recent years. In order to reduce the number of repeaters in the power distribution system, Ahmed et al. [35] combine customers and distributors into one domain and improves network performance with a limited number of smart relays. In terms of power distribution, Ma et al. [6] utilize a collaborative learning multi-swarm artificial bee colony algorithm to search for the optimal value of power distribution systems. Kilkki et al. [36] propose an agent-based model for simulating different smart grid frequency control schemes. While communication-based models also play a role in power regulation stability, Li [1] studies cooperative communication to improve the reliability of transmission in smart grid networks. Yu et al. [37] propose a novel distributed control strategy based on a back-and-forth communication framework to optimally coordinate multiple energy storage systems for multi-time-step voltage regulation. Although many achievements have been made in distributed power regulation, and the research on communication in distributed power has made great progress, there is still little research on how to reduce communication in cooperative grid systems. Therefore, our work mainly focuses on how to reduce the communication between distributed grid nodes while ensuring system stability.

### 2.2. Communications in Cooperative Agents

Communication, which is the process of agents sharing observations and experiences [38], is the bridge of cooperation in multi-agent systems. In collaborative multi-agent reinforcement learning, much attention has been paid to how to learn more effective communication strategies. Foerster et al. [22] propose differentiable inter-agent learning claim that agents can back-propagate error derivatives through communication channels, which share the gradients between different agents to learn effective communications mechanism. Hoshen [39] proposes attentional multi-agent predictive modeling, which is an attention vector for selecting helpful agents to interact with, achieving a better performance than the average pooling method [23]. ATOC [40] extends the methods through local interaction. Das et al. [25] propose targeted multi-agent communication that distinguishes the importance of a message and transmits it to different agents. Recently, there have been some attention-based methods to infer the relationship between agents and guide an agent to send messages to the most relevant agents [17,41,42,43,44,45]. These algorithms have made many improvements in the way of communication utilization to improve the effect of collaboration. However, every time the agent system communicates, all information exchange between agents is required, which does not essentially reduce the unnecessary communication bandwidth. Therefore, our method focus on letting each agent selectively send important information to reduce communication bandwidth.

### 2.3. Causal Inference

Causal inference plays a significant role in improving the correctness and interpretability of deep learning systems [46,47,48]. Researchers focus on using causal inference in the reinforcement learning community to mitigate estimation errors of off-policy evaluation in partially observable environments [49,50,51]. Bibaut et al. [49] use the maximum likelihood estimation principle of causal inference objectives to study the problem of off-policy evaluation in reinforcement learning. Tennenholtz et al. [50] focus on the second level of causal theory, hoping to know the effects of using strategy to intervene in the world and take different actions. Oberst and Sontag [51] focuse on the counterfactual and studies what happens in a specific trajectory under different strategies with all other variables (including random noise variables) being the same. Besides, causal inference helps enhance the interpretability of the machine learning model in online learning and bandit methods [52,53,54,55]. Jaber et al. [52] provide a method to learn policy values from off-policy data based on state and action using a latent variable model. Lu et al. [55] take the confounding variant into account, solves the complete reinforcement learning problem with the observed data, and puts forward the general formula of the reinforcement learning problem.

The causal inference in single-agent reinforcement learning has been extensively studied. However, the research on the communication effect of reinforcement learning agents using causal inference is relatively few. Our research establishes a generalized reinforcement learning formula with communication similar to [55]. With causal inference, we focus on the different effects of communication intervention on agents.

## 3. A Graphical Model: Preliminaries

Transforming the distributor cooperation problem in smart grids into a multi-agent cooperation problem facilitates our modeling and better problem definition. In multi-agent and collaborative agents systems, not all of the communications are necessary and some of them will even degrade model performance [17,26]. Therefore, an appropriate communication selection mechanism can not only promote cooperation among agents, but also can reduce communication bandwidth. As a mechanism to reduce communication, causal inference can evaluate the impact of communication by the rewards of agents, so that agents can determine whether an communication sending is necessary and beneficial at current state. Therefore, in this section, we first introduce the definitions of the causal model and then elaborate the use of graphical models to represent reinforcement learning.

### 3.1. Causal Models

In a smart grid system, the difference in the effect of distributors communication or non-communication can be predicted by estimating ITE. Estimating ITE, which is defined as the expected difference between treatment and control outcomes, is one of the most challenging in causal inference [30,31]. An individual belongs to either the treatment group or the control group. The entire vector of potential outcomes can never be obtained exactly because only factual outcomes can be revealed by experiment results, and counterfactual outcomes cannot be accessed directly. One way to tackle this issue is to estimate counterfactual outcomes from visible observation data. Let us first sort through some definitions of the identification of causal inference.

**Definition** **1.** *Graphs as Models of Interventions*

*Probability model is used to describe the relationship between variables in smart grid system. Compared to other probabilistic models, causal models can be used to estimate the effect of interventions. This additional feature requires the joint distribution to be complemented with a causal diagram, i.e., a directed acyclic graph (DAG) G that identifies the causal connections between variables [56,57,58], which is called Bayesian networks. Based on the Bayes theorem, we can decompose the joint distribution into a child-parent relation form as DAG. Then, by leveraging the chain rule of probability calculus, we formulate the conditional distribution as:*

(1) $$P\left(x_{1},\cdots ,x_{n}\right)=\prod_{j}P\left(x_{j}|pa_{j}\right)$$

*where $x_{j}$ is vertices (nodes) and $pa_{j}$ is the parent node of $x_{j}$.*

**Definition** **2.** *Individual Treatment Effect (ITE)*

**Figure 1:**
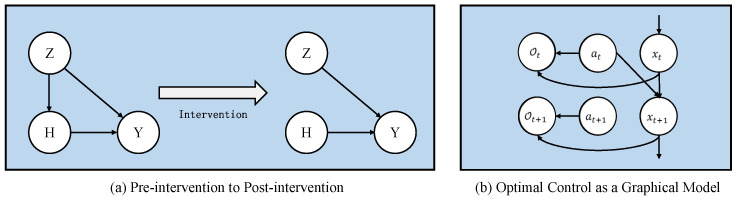
Back-door criterion and graphical model of optimal control.

*As shown in Figure 1a, ITE estimation is used to measure the effect of outcome $Y_{h}$ by a treatment h in causal graph G. In the case of a binary action set h={0,1}, where action 1 represents “treatment” and action 0 is “control”. ITE are formulated as $ITE=Y_{1}-Y_{0}\;$, where $Y_{0}$ is the potential outcome of treatment h=0, and $Y_{1}$ indicates the potential outcome of treatment h=1. In this work, we construct our objective using notation from probability theory. Z; H; Y are random variables, and z; h; y are instances of random variables.*

**Definition** **3.** *Back-door Criterion*

*The pre- and post-intervention processes are shown in Figure 1a. After intervention, the edge pointing to the intervention variable will be removed, and the data distribution will change accordingly. Calculating the impact of interventions on outcomes requires back-door criterion. A set of variables Z satisfies the back-door criterion relative to an ordered pair of variables (H,Y) in a DAG G if (1) no node in Z is a descendant of the intervention variable H; and (2) Z blocks every path between H and Y that contains an arrow into Y. The Back-door adjustment formula is written as:*

(2) $$P\left(Y=y|do\left(H=h\right)\right)=\sum_{z}P\left(Y=y|H=h,Z=z\right)P\left(Z=z\right)$$

*where the do(·) means to apply an intervention on the variable.*

### 3.2. Reinforcement Learning as a Graphical Model

In smart grid systems, the strategy of distributors is obtained using reinforcement learning algorithms. Describing reinforcement learning as a graphical model can facilitate us to relate the graphical model with a causal inference. Therefore, this subsection integrates the notation of the causal model into the reinforcement learning (RL) formulation. At time step *t*, the state and action are $x_{t}$ and $a_{t}$, respectively. States, actions, and the next states are represented by nodes in the graph model and linked by relational edges. The state transition can be expressed as $p(x_{t+1}|x_{t},a_{t})$, which can be viewed as the influence relationship between state and action. In the state transition process, the environment will feed back the agent with a reward *r*, which cannot be directly expressed in the graph model. A binary variable $O_{t}$ is introduced to indicate whether an action is optimal: $O_{t}=1$ means action $a_{t}$ is optimal at time step *t* given state $s_{t}$ and $O_{t}=0$ means action $a_{t}$ is not optimal. The probability distribution of $O_{t}$ is $p(O_{t}=1|x_{t},a_{t})$, which has an exponential relationship with the reward $r(x_{t},a_{t})$ as follows:

(3) $$p\left(O_{t}=1|x_{t},a_{t}\right)=\exp\left(r\left(x_{t},a_{t}\right)\right)$$

In the following, for convenience, we will remove = 1 from the rest of the derivation and default to $O_{t}=1$, $p\left(O_{t}|x_{t},a_{t}\right)=\exp\left(r\left(x_{t},a_{t}\right)\right)$. The graph model after adding node representing random variable $O_{t}$ is represented in Figure 1b. By optimizing this distribution $p(O_{t})$, via approximate inference, we can get an objective function of maximum entropy [59].

## 4. Method: The Causal Inference Communication Model (CICM)

The challenge of distributor cooperation in smart grids is a natural multi-agent collaboration problem. In order to reduce the communication frequency in smart grids and further reduce the communication bandwidth, we propose the CICM. This section describes CICM in detail. We first discuss the reinforcement learning with the causal model and establish a graphcial model, which offers a flexible framework. Then, based on the graphcial model, we introduce ITE to determine whether agents need to send communication or not.

### 4.1. Connecting Causal Model and Reinforcement Learning

**Figure 2:**
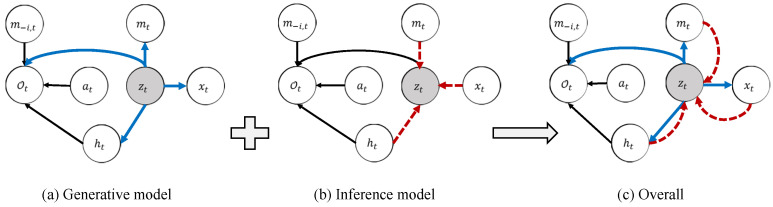
Graphical model with latent variable model. In (**a**), the solid blue arrows represent the generative model $p_{\theta }\left(z_{t}\right)p_{\theta }\left(x_{t}|z_{t}\right)p_{\theta }\left(m_{t}|z_{t}\right)p_{\theta }\left(h_{t}|z_{t}\right)$. In (**b**), the dashed red arrows denote the variational approximation $q_{\phi }\left(z_{t}|x_{t},m_{t},h_{t}\right)$. $m_{-i,t}$ is the message that comes from other agents. The overall computational paths of CICM is shown in (**c**).

Strategies for distributors in a smart grid can be learned using reinforcement learning, and the communication strategies between distributors can be obtained using causal inference. To connect them, we integrate the graphical model of reinforcement learning and causal inference into a single graphical model. The reinforcement learning embedded into the graphical model is shown in Figure 1b. The objective function can be obtained with maximum entropy [59] through approximate inference. To integrate with the causal model, this paper extends the graphical model of reinforcement learning by introducing a latent variable *z* and an intervention variable *h*, as shown in Figure 2. In the smart grid systems, the intervention variable *h* refers to whether the distributor node accepts external messages. The latent variable *z* adopts variational autoencoder (VAE) to learn a state representation vector in control problem. Through VAE, we can obtain an informative supervision signal. A latent vector *z* representing any uncertainty state variables is quickly learned during training. The intervention variable *h* controls the presence or absence of communication. The agent *i* accepts $m_{-i}$ from the oracle agent when h=1, and rejects $m_{-i}$ when h=0, where the *m* is the communication of agent *i* and the $m_{-i}$ is the communication from other agents. The intervention variable allows us to employ a causal model to estimate the impact of communication on the distribution $O_{t}$.

Figure 2 presents the probabilistic graphical model, containing the latent variable $z_{t}$, the agent’s observation data *x*, intervention variable *h*, outgoing communication *m* and communication from other agents $m_{-i}$, and action *a*. We first use the chain rule to express the relationship between the variables with the Bayesian formula:

(4) $$\begin{array}{cc} \; & p_{\theta }\left(x_{t},O_{t},z_{t},a_{t},h_{t},m_{t}\right)=p_{\theta }\left(x_{t}|z_{t}\right)p_{\theta }\left(m_{t}|z_{t}\right)p_{\theta }\left(z_{t}\right)p_{\theta }\left(O_{t}|z_{t},a_{t}{,h}_{t}\right)p_{\theta }\left(a_{t}\right)p_{\theta }\left(h_{t}|z_{t}\right) \end{array}$$

The variational distribution of a graph model can be written as the product of recognition terms $q\left(z_{t}|x_{t},a_{t},h_{t}{,m}_{t}\right)$ and policy terms $\pi (a_{t}|x_{t},h_{t},m_{-i,t})$:

(5) $$\begin{array}{c} q\;\left(z_{t},a_{t}\;|x_{t},h_{t},m_{t}\right)\;=\;q\left(z_{t}|x_{t},a_{t},h_{t}{,m}_{t}\right)\;\pi \left(a_{t}|x_{t},h_{t},m_{-i,t}\right) \end{array}$$

Optimizing the evidence lower bound (ELBO) can obtain the maximum marginal likelihood $\log p(x_{t},O_{t},h_{t},m_{t})$ [59]. From the posterior of the variational distribution (Equation (5)) and the likelihood of the joint distribution Equation (4), whose marginal likelihood can be soloved by Jensen’s inequality. The ELBO is:

(6) $$\begin{array}{c} \log p\left(x_{t},O_{t},h_{t},m_{t}\right)=D_{\mathrm{KL}}\;\left(q\left(z_{t}\;,a_{t}\;|x_{t},h_{t},m_{t}\right)\;\left|\right|p\left(z_{t},a_{t}|\;x_{t},h_{t},m_{t},O_{t}\right)\right)\;+\;\mathrm{ELBO} \end{array}$$

The first term of the above equation (Equation (6)) is the KL-divergence of the approximate from the true posterior. Since $\log p\left(x_{t},O_{t},h_{t},m_{t}\right)$ is fixed and KL-divergence is positive, we convert the problem into optimizing ELBO [32]:

(7) $$\begin{array}{c} \mathrm{ELBO}\;=\;-D_{\mathrm{KL}}\left(q\left(z_{t},a_{t}|x_{t},h_{t},m_{t}\;\right)\;\right|\;\left|\;p\left(z_{t},a_{t}\right)\right)\;+\;\;E_{z_{t},a_{t}\;∼q\left(\cdot |x_{t},h_{t},m_{t}\;\right)}\;\left[\log p\left(\;x_{t},h_{t},m_{t},\;O_{t}|z_{t},a_{t}\;\right)\right] \end{array}$$

We rewrite the ELBO as follows, and present the complete derivation of the ELBO in Appendix A.

(8) $$\begin{array}{c} \underset{\mathrm{latent}\;\mathrm{variable}\;\mathrm{model}\;\mathrm{terms}}{\underbrace{\begin{array}{cc} \; & \mathrm{ELBO}\\ = & E_{a_{t}∼\pi \left(\cdot |x_{t},h_{t},m_{-i,t}\right)}\left[E_{z_{t}∼q\left(\cdot |x_{t},a_{t},h_{t}{,m}_{t}\right)}\left(\log p_{\theta }\left(x_{t}|z_{t}\right)+\log p_{\theta }\left(h_{t}|z_{t}\right)+\log p_{\theta }\left(m_{t}|z_{t}\right)\right)\right]\\ \; & +E_{a_{t}∼\pi \left(\cdot |x_{t},h_{t},m_{-i,t}\right)}[-D_{\mathrm{KL}}\left(q_{\phi }\left(z_{t}|x_{t},a_{t},h_{t}\right)|\left|p_{\theta }\left(z_{t}\right)\right)\right] \end{array}}} \end{array}$$

(9) $$\begin{array}{c} \underset{\mathrm{maximum}\;\mathrm{entropy}\;\mathrm{objective}\;\mathrm{terms}}{\underbrace{+\;E_{a_{t}∼\pi \left(\cdot |x_{t},h_{t},m_{-i,t}\right)}\left[E_{z_{t}∼q\left(\cdot |x_{t},a_{t},h_{t}{,m}_{t}\right)}\left(r\left(z_{t},a_{t}{,h}_{t},m_{-i,t}\right)+H\left(\pi \left(a_{t}|x_{t},h_{t},m_{-i,t}\right)\right)\right)\right]}} \end{array}$$

where $r\left(z_{t},a_{t}{,h}_{t},m_{-i,t}\right)=\log p_{\theta }\left(O_{t}|z_{t},a_{t}{,h}_{t}\right)$ in control as inference framework. For simplicity, we omit the constant term, i.e., uniform action prior $\log p(a_{t})$, in ELBO. The first term Equation (8) of the ELBO is the latent variable model about latent variable *z*, which is marked with $J_{M}\left(\theta ,\phi \right)$. Besides, there are generative model $p_{\theta }\left(x_{t}|z_{t}\right)$, $p_{\theta }\left(h_{t}|z_{t}\right)$ and $p_{\theta }\left(m_{t}|z_{t}\right)$, as well as inference model $q_{\phi }\left(z_{t}|x_{t},a_{t},h_{t}\right)$. The second term Equation (9) is the maximum entropy objective function [59].

**Figure 3:**
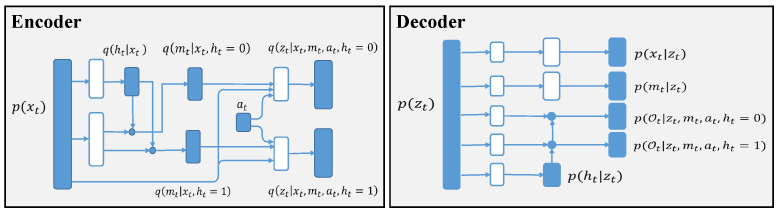
Architecture of the model and inference networks. White nodes are the neural network, blue nodes are samples from the distribution, and blue dots are switches according to the treatment *h*.

Figure 3 shows the architecture of the model and inference network, which includes the encoder and decoder in the VAE.

### 4.2. Estimating Individual Treatment Effect

ITE is used to compute the difference in outcomes caused by intervention variable *h*. In actual cases, only the outcomes caused by treatment or control can be observed, and the counterfactual outcomes of unimplemented interventions are always missing. Similarly, during the training of RL agents, only the outcomes of specific communication choice are observed. We cannot obtain the individual-level effect directly from the observed trajectories of agent. The immediate challenge is to estimate the missing counterfactual outcomes based on the historical trajectories and then estimate the ITE.

According to Definition 2, the ITE $C_{\mathrm{ITE}}$ of an agent on the intervention variable *h* is as follows. It is measured as the difference between the expected treatment effect when h=1 (accept $m_{-i}$ from the other agents) and h=0 (reject $m_{-i}$ from the other agents), which can be written as:

(10) $$C_{\mathrm{ITE}}=p_{\theta }\left(O_{t}|z_{t},a_{t},m_{-i,t},do\left(h_{t}=1\right)\right)-p_{\theta }\left(O_{t}|z_{t},a_{t},m_{-i,t},do\left(h_{t}=0\right)\right)$$

The $do(h_{t}=1)$ in the above formula refers to the intervention condition $h_{t}=1$. According to the backdoor criterion in Definition 3, we apply the rules of the do-calculus to Figure 2. We can get:

(11) $$\begin{array}{cc} \; & p_{\theta }\left(O_{t}|x_{t},a_{t},m_{-i,t},do\left(h_{t}=1\right)\right) \end{array}$$

(12) $$\begin{array}{cc} = & \int_{z_{t}}^{}p_{\theta }\left(O_{t}|x_{t},z_{t},a_{t},m_{-i,t},do\left(h_{t}=1\right)\right)p_{\theta }\left(z_{t}|x_{t},a_{t},m_{-i,t},do\left(h_{t}=1\right)\right)d_{z_{t}} \end{array}$$

(13) $$\begin{array}{cc} = & \int_{z_{t}}^{}p_{\theta }\left(O_{t}|x_{t},z_{t},a_{t},m_{-i,t},h_{t}=1\right)p_{\theta }\left(z_{t}|x_{t},a_{t},m_{-i,t}\right)d_{z_{t}} \end{array}$$

(14) $$\begin{array}{cc} = & \int_{z_{t}}^{}p_{\theta }\left(O_{t}|z_{t},a_{t},m_{-i,t},h_{t}=1\right)p_{\theta }\left(z_{t}|x_{t},a_{t},m_{-i,t}\right)d_{z_{t}} \end{array}$$

(15) $$\begin{array}{cc} = & E_{z_{t}∼p_{\theta }\left(\cdot |x_{t},a_{t},m_{-i,t}\right)}\left[p_{\theta }\left(O_{t}|z_{t},a_{t},m_{-i,t},h_{t}=1\right)\right] \end{array}$$

where the transition from (12) to (13) is by the rules of do-calculus applied to the causal graph in Figure 2. The $O_{t}$ and $x_{t}$ are independent of each other under the condition given by $z_{t}$, $O_{t}⊥x_{t}|z_{t}$, which transforms the formula from (13) to (14). Similarly, $p_{\theta }\left(O_{t}|x_{t},a_{t},m_{-i,t},do\left(h_{t}=0\right)\right)=E_{z_{t}∼p_{\theta }\left(\cdot |x_{t},a_{t},m_{-i,t}\right)}\left[p_{\theta }\left(O_{t}|z_{t},a_{t},m_{-i,t},h_{t}=0\right)\right]$.

We can obtain the ITE $C_{\mathrm{ITE}}$ in probabilistic form. The following formula can be calculated using the data distribution before the intervention. We use the backdoor criterion to estimate the ITE in the following form:

(16) $$C_{\mathrm{ITE}}\;=\;E_{z_{t}∼q_{\phi }\left(\cdot |x_{t},a_{t},m_{-i,t}\right)}\;\left[p_{\theta }\left(O_{t}|z_{t},a_{t},m_{-i,t},h_{t}=1\right)-p_{\theta }\left(O_{t}|z_{t},a_{t},m_{-i,t},h_{t}=0\right)\;\right]\;$$

The following formula is used to determine the value of the binary variable $h_{t}$ based on the prediction result of $C_{\mathrm{ITE}}$. The value of the binary variable $h_{t}$ refers to whether the agent needs to communicate.

(17) $$h_{t}=\left\{\begin{array}{cc} 1, & \;\mathrm{if}\;\;C_{\mathrm{ITE}}>0\\ 0, & \;\mathrm{if}\;\;C_{\mathrm{ITE}}\leq 0 \end{array}\right.$$

We add a term to the latent variable model $J_{M}\left(\theta ,\phi \right)$ to help us predict $p_{\theta }\left(O_{t}|z_{t},a_{t},m_{-i,t},h_{t}\right)$.

(18) $$J=J_{M}\left(\theta ,\phi \right)+E_{z_{t}∼q_{\phi }\left(\cdot |x_{t},a_{t},m_{-i,t}\right)}\left[\log p_{\theta }\left(O_{t}^{*}|z_{t},a_{t},m_{-i,t},h_{t}=h_{t}^{*}\right)\right]$$

Here, $O_{t}^{*}$,$h_{t}^{*}$ are the actual observations. We use the relationship between the optimal distribution variable $O_{t}$ and the reward *r*, $p_{\theta }\left(O_{t}|z_{t},a_{t},m_{-i,t},h_{t}\right)=\exp\left(r\left(z_{t},a_{t},m_{-i,t},h_{t},\right)\right)$ to calculate the label value corresponding to the distribution.

## 5. Experiments, Datasets and the Environment

We first introduce the power grid problem, a distributed distributors environment in smart grid. In addition, in order to fully prove the effectiveness of our method, we introduce StarCraft II and 3D environment habitation experiments. Both the Starcraft II and 3D environment habitation experiments have high-dimensional observation spaces, and they can validate the generalization ability of our model.

### 5.1. Datasets and Environment

Power Grid Problem

We test our algorithm in power grid experiments [12]. To facilitate the modeling and preliminary investigation of the problem, the simulation environment is not a common alternating current (AC) power grid network, but a direct current (DC) variant power grid network. Although the physical behavior of AC grids is quite different from that of DC grids, the method is suitable for a general learning scheme for distributed control problems.

**Figure 4:**
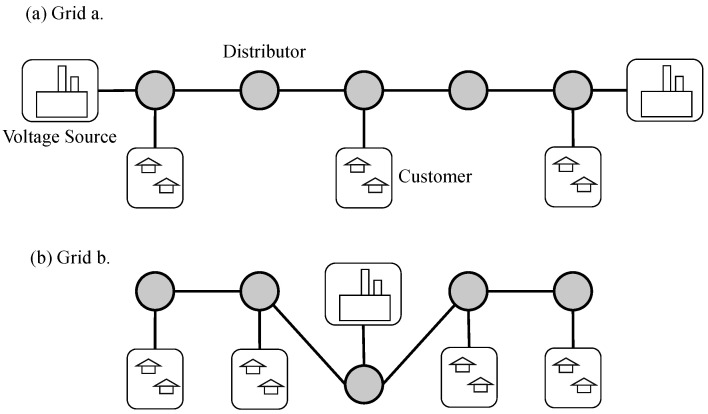
Power-grid environment. The grey circles are distributors, and their role is to distribute the voltage from the voltage source to the customer nodes.

As shown in Figure 4, the regulation system involves three parts, voltage source, city, and distributor. The grey circles are the distributors, which we model as agents in the reinforcement learning algorithm. This allows it to interact with the environment, receive observations from the environment, perform actions to adjust voltages based on the observations, and then receive reward value feedback from the environment. We set the reward value fed back to the agent by the environment as the degree to which the distributor satisfies the city’s electricity consumption. If the voltage obtained by the city node is lower than the expected value, the environment feeds back a penalty value to the distributor connected to the city node. The cooperation of multiple distributors is required to divide the voltage reasonably in the city to meet the urban electricity demand. A distributor that is not connected to a city will reward the signal with 0.

The simulated power grid problem is solved using the reinforcement learning algorithm, where the action, state, and reward values are as follows:

(1) Local actions: The power grid system controls the current by controlling the variable resistance. Each distributor node can make a choice for the power line (variable resistance) connected to it. A distributor can perform three actions on the resistance, Same, Halved, and Doubled. If a line is connected to two distributors at the same time, it is affected by both distributors at the same time, and the final selection is performed according to Table 1.

(2) Local state: The distributors receive state information from the lines connected to it. There are three types of connections for power lines: distributor-distributor, distributor-city, and distributor-voltage source. (1) Distributor-distributor: ➀ Whether it is higher than the neighbor voltage; ➁ The neighbor voltage changes, increasing, decreasing, or unchanged; ➂ The state of the resistance (maximum value, minimum value, or intermediate value) (2) Distributor-city: ➀ Whether the voltage is higher than the neighbors; ➁ Whether the city needs to increase the voltage; ➂ The state of the resistance. (3) Distributor-voltage source: ➀ Whether the voltage is higher than that of the neighbor; ➁ The state of the resistance.

(3) Local reward: When the city voltage connected to the distributor node is lower than the expected level, the environmental feedback a negative reward value to the distributor, and the reward value is equal to the difference between the actual city voltage and the expected voltage, Otherwise, the reward is 0. A distributor that is not connected to a city has a reward of 0.

Habitat

We use the multi-object navigation (multiON) dataset [60], which is adopted by artificial intelligence habitat simulators [61]. This dataset is designed for navigation tasks with the following essential elements: agent start position, direction, and target position. Eight cylindrical target objects with different colours are set. The agents’ episodes are generated based on the Matterport3D [62] scene. The data is split according to the standard scene-based Matterport3D train/val/test split [60]. Based on the multiON dataset, a multi-target sequential discovery task is constructed. The agent needs to navigate to the designated target locations in sequence in an episode. The FOUND action is defined as finding the target, which should be taken when the agent is within a distance threshold to the target. If the FOUND action is called beyond the threshold, the game fails. If the target is not found within a specified limit of the number of steps, the game is also judged as a failure. We use m-ON to denote an episode with m sequential objects. In the task, we define two heterogeneous agents. One is an oracle agent $A_{O}$ with a god perspective, and the other is an embodied navigator $A_{N}$, which performs specific navigation tasks and interacts with the environment. $A_{O}$’s observations are the position, orientation of $A_{N}$, the global information of the map, and the target position. $A_{N}$ only observes self-centered visual images with depth information. If obstacles block the target position, $A_{N}$ cannot perceive the target position and needs additional information from $A_{O}$. There is limited communication bandwidth for guidance information between $A_{O}$ and $A_{N}$. Two agents share the same reward, so they must learn to cooperate to optimize their mutual reward together.

StarCraft II Environment

**Figure 5:**
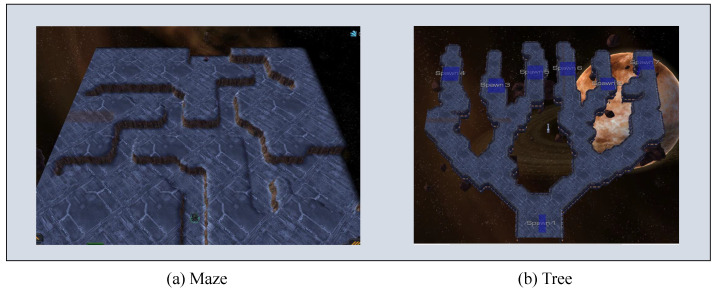
Starcraft II environment.

We design an experiment with two scenarios based on the StarCraft II environment [63], as shown in Figure 5. One is a maze task as shown in Figure 5a, and the other is a tree task of depth search as shown in Figure 5b. In the maze task, the navigator agent starts from the bottom centre point and aims to navigate to a target point whose position is randomly initialized on the road of the maze. It is noticed that the navigator agent does not know the target position. The oracle agent, on the contrary, has a god perspective that captures the target position. The oracle agent could send the relative target position (i.e., the target’s relative position to the navigator agent itself) information to the navigator agent. In the tree environment, the target point is initialized at a random leaf node in the map. Similarly, the oracle agent can pass the relative position of the target point to the navigator. We set two different depths in this scenario, depths 3 and 4. An increase in depth improves the game’s difficulty. An enemy agent is used to represent the target point for convenience. This enemy agent at the target point is inactive and has very little health, which a single attack can kill. The death of the enemy agent indicates the navigator agent successfully arrives at the target point, and the navigator agent will receive additional rewards.

### 5.2. Reward Structure and Evaluation Indicators

In Habitat and StarCraft II environment, the reward value is designed as: $r_{t}=1_{[\text{reached-goal}]}\cdot r_{\mathrm{goal}}+r_{\mathrm{closer}}+r_{\text{time-penalty}}+1_{\left[\text{send-message}\right]}\cdot r_{\text{comm-penalty}}$, where, $1_{[\text{reached-goal}]}$ is an indicator variable that takes value 1 when the navigator agent finds the current goal at time *t*. If the target is found, the agent receives reward $r_{\mathrm{goal}}$. $r_{\mathrm{closer}}$ is the difference in the distance towards target position between time step *t* and t−1. $r_{\text{time-penalty}}$ is the penalty received by the agent at time step t. There is a communication penalty $r_{\text{comm-penalty}}$ for a message sending at step *t*. To compare our results with previous studies, the communication penalty is only used in training and is excluded from the total reward in testing. In the power grid problem, our reward value is defined as the degree to which a distributor satisfies the city’s electricity consumption. If the voltage obtained by the city node is lower than the expected value, the environment feeds back a penalty value $r_{\text{power-penalty}}$ to the voltage divider connected to the city node. Like the previous two environments, the penalty reward $r_{\text{comm-penalty}}$ for communication is set in the power grid experiments.

We use the evaluation metrics in [64] on navigation tasks. In multiON [60], these metrics are extended to navigation tasks with sequential targets. We adopt the following two metrics in our experiments: PROGRESS indicates the fraction of target objects found in an episode, and PPL refers to the progress weighted by the path length.

### 5.3. Network Architecture and Baseline Algorithm

Similar to CoMON [19], CICM adopts the network structure of TBONE [65,66]. In Habitat and StarCraft II environment, $A_{O}$ encodes the information into a vector, containing the location of navigation agent $A_{N}$, the map information, and the target location. During the encoding process, $A_{O}$ in the habitat environment crops and rotates the map to construct a self-centered map, implicitly encoding $A_{N}$’s orientation into the cropped map. Then, the initial belief of $A_{O}$ is obtained through CNN and linear layers. This belief is encoded as a communication vector and sent to $A_{N}$ [65,66]. In Starcraft II, $A_{O}$ encodes inaccessible areas’ surrounding terrain and information. This information contains the target agent position and will be sent to $A_{N}$.

For Habitat environment, we use the algorithms in CoMON [19] as our comparing baselines, that are NoCom (i.e., “No communication”), U-Comm (i.e., “ Unstructured communication”), and S-Comm (i.e., “Structured communication”). We design our algorithm with a causal inference communication model based on these baseline algorithms, which are U-Comm&CIC (i.e., “U-Comm with Causal Inference model”) and S-Comm&CIC (i.e., “S-Comm with Causal Inference model”).

For the StarCraft II environment, we design the following algorithms. To meet the maximum entropy item in the ELBO model, we use the SAC [67] algorithm. Inspired by the SLAC algorithm [68], the latent variable is used in the critic to calculate the Q function $Q(z_{t},a_{t})$, and the state input $x_{t}$ of the agent is used to calculate the policy $\pi (a_{t+1}|x_{t},a_{t},h_{t},m_{-i,t})$ during execution. We design the following algorithms: SACwithoutComm, model without communication; SACwithComm, only using SAC algorithm with the communication; SACwithLatentAndComm, adding latent variable model and communication using VAE to SAC algorithm; CICM (our method), leveraging causal inference and VAE model.

For the power grid environment, unlike the previous two environments, the power network involves communication between multiple agents. When the agent sends information, it is also the receiver of information. We encode the information sent by the distributors connected to the receiver, and finally take the average value as the received information. The algorithm design in the power grid network is the same as that used in StarCraft II environment.

## 6. Analysis of Experiment Results

In this section, we first analyze the computational complexity of CICM, and then analyze the performance of the algorithm in three experimental environments (power grid, StarCraft, and habitat).

### 6.1. Complexity Analysis

We theoretically analyze the complexity of our algorithm CICM. In the smart grid environment, we consider that all agents can communicate with each other, so that they can form a complete graph. If there are *N* agents, the computational complexity is $O(N^{2})$. If there are no more than ω neighbor nodes, our computational complexity is less than O(ωN). Therefore, the computational complexity is acceptable.

In the neural network, the computational complexity of our algorithm is related to updating parameters during training. Use *U* to represent the total number of training episodes. In each episode, there are *T* steps. We set the computational complexity of the ITE module to *M* and the computational complexity of the reinforcement learning (SAC) to *W*. During training, the update is made every *C* steps. The computational complexity of our algorithm is O(UT(M+W)/C). We define $D_{x}$ is the dimension of states, $D_{m}$ is the dimension of communication, $D_{n}$ is the dimension of hidden layer, $D_{z}$ is the latent variable dimension, $D_{a}$ is the action dimension, and the binary variable dimension $D_{h}$ and $D_{O}$ is 1. In ITE, there are two modules, including encode and decoder. In the encoder module, the computational complexity is $O(D_{x}D_{n}D_{h}+\left(2D_{x}D_{h}+D_{x}D_{n}D_{m}\right)+2\left(D_{x}+D_{m}+D_{a}\right)D_{n}D_{z})$. The computational complexity of the decoder module is $O(2D_{z}D_{n}D_{x}+2D_{z}D_{n}D_{m}+2\left(D_{z}+D_{m}+D_{a}\right)D_{n}D_{O}+D_{z}D_{n}D_{h})$. The reinforcement learning (SAC) also includes two modules. The computational complexity of Critic is $O(2D_{z}D_{n}D_{a})$, which is involving two Critic. The calculation complexity in Actor is $O(\left(D_{x}+\omega D_{m}+D_{a}+D_{h}\right)D_{n}D_{a})$.

### 6.2. Power Grid Environment

**Figure 6:**
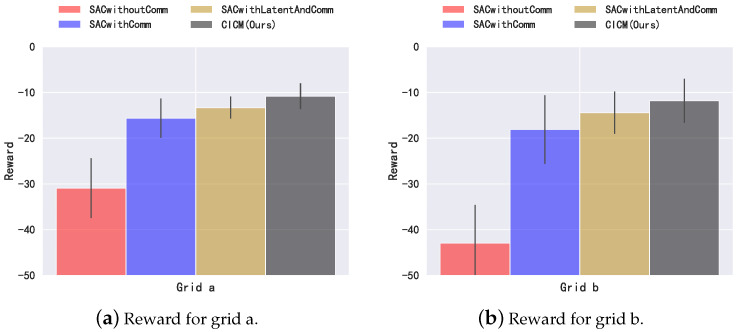
Return in the power grid environments. Error bars are one standard deviation. The communication penalty is set to $r_{\text{comm-penalty}}=-0.1$.

Our algorithm has a communication penalty in the reward during training, and for a fair comparison, we do not calculate the penalty during testing. Figure 6 shows the penalty values under two different grid structures. Table 2 shows the communication probability of our algorithm CICM, which is calculated by dividing the number of time steps of communication by the number of communication steps of the full communication algorithm (communication is carried out at each time step). In Figure 6a,b, we can see that the algorithm without communication, SACwithoutComm, receives significantly more penalties than the other three algorithms with communication. Among the three algorithms with communication, the algorithm SACwithComm, which directly uses communication, is better than the algorithm without communication. However, SACwithComm is not as high as the algorithm SACwithLatentAndComm which combines the latent variable model in the utilization of communication information. Our algorithm CICM, a communication model that combines latent variable model and causal inference for communication judgment, shows the best performance. The judgment of communication helps the agent filter unnecessary information, which reduces the penalty caused by the distribution voltage while reducing the communication cost.

We further analyze the communication probability in Table 2. Since we set the communication penalty $r_{\text{comm-penalty}}$, as long as the agents communicate with each other, the system will receive the communication penalty, and the communication penalty is included in the feedback reward of the system. The application of the communication penalty will reduce the feedback reward, which reduces the probability of the system getting optimal feedback, $p_{\theta }\left(O_{t}|z_{t},a_{t},m_{-i,t},h_{t}\right)$. In order to increase the probability of optimal feedback, the system needs to reduce the communication probability, thereby reducing the communication penalty and increasing the probability of the system getting the optimal feedback. We test our model on grid a (Figure 4a) and grid b (Figure 4b) and obtain 37.4% and 32.9% communication probability, respectively. The experiment shows that our model uses a small number of communications to reduce the penalty value. However, the communication probability won’t be reduced to zero, since the power grid system requires certain communication to ensure cooperation among distributors.

### 6.3. StarCraft Experiment

**Figure 7:**
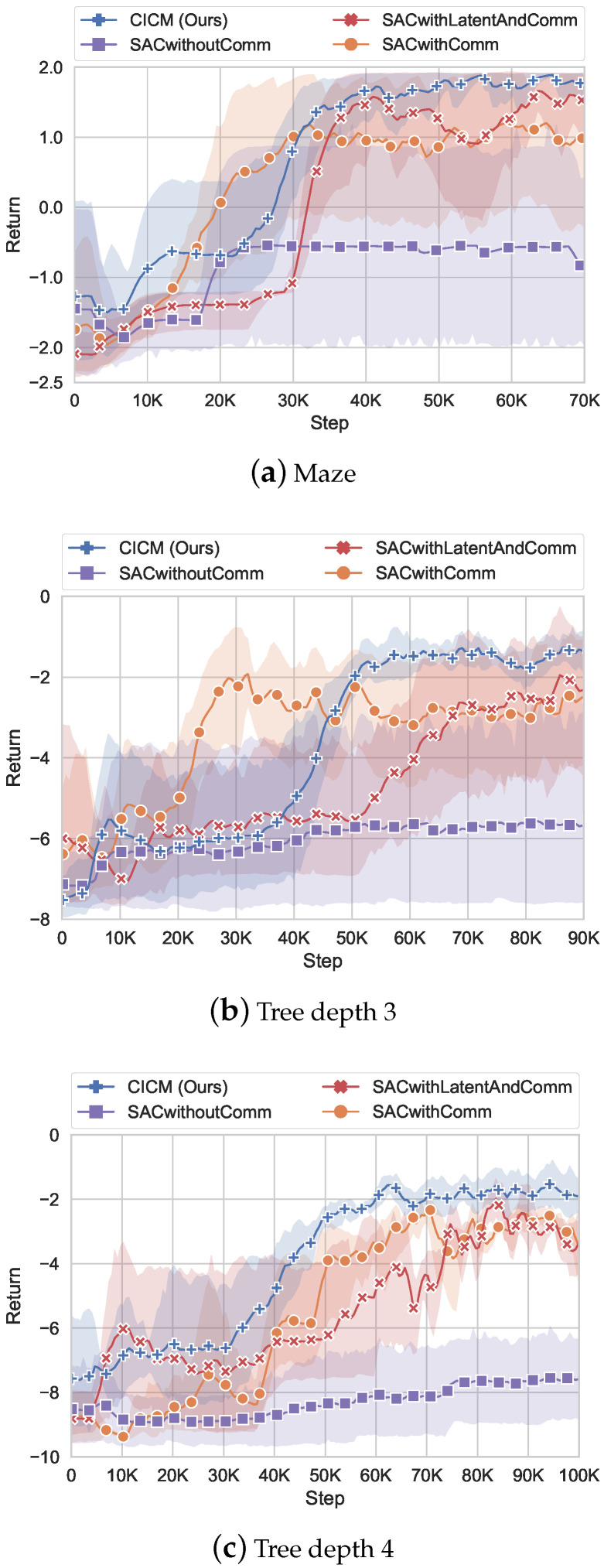
Return in the StarCraft environment. Error bars are one standard deviation over 6 runs. The communication penalty is set to $r_{\text{comm-penalty}}=-0.5$.

Figure 7 shows the reward for our StarCraft II environment. There is a communication penalty in the reward in our algorithm during training, and we do not count the penalty generated by communication costs during testing for a fair comparison. All the graphs show that the reward learned by algorithms without communication is significantly lower than the algorithms with communication. This is because the oracle agent provides critical information about navigation, which includes the target position. We can see that the convergence speed of SACwithComm is fast, and it rises quickly in all of the three graphs at the beginning. In contrast, the models with latent variables (CICM and SACwithlatentAndcomm) have a slow learning speed initially. Because a certain amount of data is required to train a stable VAE model. After obtaining a stable VAE, the SACwithlatent algorithm rises rapidly, surpassing the performance of SACwithComm on the maze and slightly exceeding the performance of SACwithComm on the tree environment. It reveals that the latent model has improved the performance of the algorithm.

CICM integrates the latent model and causal inference for communication judgment. With the help of the latent model, even although our algorithm learns slowly at the beginning (Figure 7b), it achieves the highest and most stable performance among all of the algorithms at the end. CICM’s final stage is higher than others because of the introduction of a communication strategy. It allows the agent to reduce unnecessary communication and memory information in the RNN network and thus obtain the most negligible variance in all three experiments. Table 3 shows the communication probability of our algorithm and test result under different single-step communication penalties in the maze environment. From the table, we can see that an increase in communication penalty will decrease communication frequency. The performance difference between different communication probability is not very large, and CICM achieves the best performance when the single-step communication penalty is −0.5. Therefore, we adopt −0.5 as the default value in the experiments.

We further analyze Table 3. From Table 3, we can see that the smaller communication penalty, the smaller the impact on communication probability. This is because the system regards the communication penalty as a part of the feedback. When the communication penalty is small, the communication penalty will not play a big role in the probability of getting the optimal feedback $p_{\theta }\left(O_{t}|z_{t},a_{t},m_{-i,t},h_{t}\right)$. But when the communication penalty becomes $r_{\text{comm-penalty}}$ = −0.5, we find that the communication probability drops significantly to 38.0%. The reason is that the communication penalty affects the probability of the system getting the optimal feedback, and the communication probability needs to be reduced to make the feedback optimal. At the same time, we can also notice that the communication penalty cannot be increased indefinitely. Although the communication penalty can make the algorithm reduce communication probability, the lack of communication will also affect the cooperation between agents. It can be seen from the table that when the communication penalty reaches −2, the communication volume is reduced to 27.4%, but at the same time, the obtained test result will also be reduced to 1.75.

### 6.4. Habitat Experiment

Below we analyze the algorithm performance in Habitat. Our algorithm is the first trained on the 3-ON setting, and then gets a stable model. We merge the counterfactual communication model based on the trained model and finally get our overall model. We test this model on 1-ON, 2-ON, and 3-ON, respectively, and the final results are presented in Table 4. We test the effect of different hyperparameters $r_{\text{comm-penalty}}$ on the communication probability of our algorithm S-Comm&CIC, as shown in Table 5.

NoCom provides our algorithm with a lower bound on what can be achieved without communication. Our algorithm adds the causal inference communication model on U-Comm and S-Comm (which we name as U-Comm&CIC and S-Comm&CIC). U-Comm&CIC and S-Comm&CIC are close to the effect of the original algorithm. At 3-ON, our algorithm slightly exceeds the original in the indicator PPL. A higher PPL metric indicates that our algorithm can successfully navigate over shorter distances.

## 7. Discussion

In this section, we mainly discuss the research limitations and the threat to validity.

### 7.1. Research Limitations

The following is our summary of the limitations of our algorithm:

- We only conduct experimental tests on the DC power grid simulation environment of the power grid, which is different from the real production environment. There is still a big gap between simulation and reality. How to deploy the algorithms trained in the simulation environment to the real environment requires more effort.
- Our algorithm only considers the counterfactual of causal inference in agents’ communication, and does not introduce some cooperative mechanisms of multi-agent reinforcement learning in policy learning, nor does it consider the credit assignment among multiple agents. The introduction of these mechanisms in the future will further improve the performance.
- Our algorithm mainly takes consideration of theoretical completeness, thus introducing latent variable models with reinforcement learning objective function and causal inference. However, the model is relatively complex, and its stability is weak during training, and the stability of the algorithm needs to be paid attention to in the follow-up research.

### 7.2. Threat to Validity

Internal validity: Our algorithm is tested in three experimental environments. In the environment of power grid and StarCraft, the reinforcement learning module is designed based on SAC algorithm. We add the ITE module to SAC to form our algorithm CICM. The ITE module is not included in the basic algorithm of comparison. We strictly follow the control variables in the parameters of the algorithm. The parameters used in all the reinforcement learning module modules are consistent. In the Habitat environment, we also add our ITE module to the basic learning reinforcement learning algorithms U-Comm and S-Comm. We do not change the parameters in the basic reinforcement learning algorithm. In summary, our experiments are carried out under the control of variables, and it is internally valid.

External validity: Our algorithm is proved to be feasible by experiments on power grid system. To prove the scalability of the algorithm, we continue to do experiments on high-dimensional game environments StarCraft and Habitat. In addition, it performs well compared with the baseline algorithm in those high-dimensional game environments. Our algorithm is extensible in different fields, so it is external validity.

## 8. Conclusions

As electricity demands are increasingly significant, it is necessary to develop a power grid system that can meet higher and more diversified market demands and is safe, reliable, and stable performance. The emerging smart grid can meet the current challenges faced by the traditional power grid system as it is based on more intelligent power distribution agents and methods to increase electricity generation and ensure safety, reliability, and stability. The distributed control of the smart grid system requires a large amount of bandwidth to meet the communication needs between distributor nodes. To ensure that the system performs well and reduces the communication bandwidth, we propose CICM, which adopts a causal inference model to help agents decide whether communication is necessary. The causal inference is constructed on a graphical model with the Bayesian formula. The graphical model connects optimal control and causal inference. Estimating the counterfactual outcomes of the intervention variables and then evaluating the ITE helps the agent make the best communication strategy. We conduct experiments on smart grid environment tasks. Our experiments show that CICM can reduce the communication bandwidth while improving performance in the power grid environment. In addition, we also conduct experiments on StarCraft II navigation tasks and the 3D Habitat environment, and the experimental results once again prove the effectiveness of the algorithm. This study serves as a first attempt to optimise control and reinforcement learning with causal inference models. We believe that the causal inference frameworks will play a more significant role in reinforcement learning problems.

Future research will focus on extending the current model in several directions, such as model multiple distributors, explicit modeling of game theoretical aspects of the graph theory modeling, distributed computing, causal modeling of other reinforcement learning agents, model-based multi-agent reinforcement learning, and variational inference for accelerating off-line multi-agent reinforcement learning.

## Figures and Tables

**Table 1:** The joint action of two distributors.

Distributor 1	Distributor 2		
	Halve	Same	Double
Halve	Halve	Halve	Same
Same	Halve	Same	Double
Double	Same	Double	Double

**Table 2:** Communication probability in power grid environment. The communication penalty is set to $r_{\text{comm-penalty}}=-0.1$.

Environment	Grid a	Grid b
Communication probability	37.4%	32.9%

**Table 3:** Communication probability and test result for different communication cost in Starcraft. Communication probability is calculated by dividing the number of time steps of communication by the number of communication steps of the full communication algorithm (communication is carried out at each time step).

Communication Penalty	−0.05	−0.15	−0.20	−0.5	−1.0	−1.5	−2.0
$r_{\text{comm-penalty}}$							
Communication probability	48.8%	45.0%	46.3%	38.0%	32.7%	29.1%	27.4%
Test Result	1.83	1.87	1.89	1.93	1.85	1.79	1.75

**Table 4:** Task performance metrics for different communication mechanisms evaluated in Habitat. The communication penalty is set to $r_{\text{comm-penalty}}=-0.001$.

	PROGRESS (%)			PPL (%)		
	1-ON	2-ON	3-ON	1-ON	2-ON	3-ON
NoCom	56	39	26	35	26	16
U-Comm	87	77	63	60	51	39
S-Comm	85	80	70	67	59	50
U-Comm&CIC	84	76	64	57	49	40
S-Comm&CIC	86	78	70	67	57	51

**Table 5:** Communication cost and the communication probability in Habitat 3-ON.

Communication Penalty $r_{\text{comm-penalty}}$	−0.001	−0.01	−0.1
Communication probability	38.3%	34.8%	29.5%

## Data Availability

Not applicable.

## Appendix A. Derivation of the Latent Variable Model

Figure 2 presents the probabilistic graphical model, containing the latent variable $z_{t}$, the agent’s observation data *x*, communication *m*, intervention variable *h*, and action *a*. We first use the chain rule to express the relationship between the variables with the Bayesian formula:

(A1) $$\begin{array}{cc} \; & p_{\theta }\left(x_{t},O_{t},z_{t},a_{t},h_{t},m_{t}\right)=p_{\theta }\left(x_{t}|z_{t}\right)p_{\theta }\left(m_{t}|z_{t}\right)p_{\theta }\left(z_{t}\right)p_{\theta }\left(O_{t}|z_{t},a_{t}{,h}_{t}\right)p_{\theta }\left(a_{t}\right)p_{\theta }\left(h_{t}|z_{t}\right) \end{array}$$

The conditional distribution of graphical model (Figure 2) can be written as:

(A2) $$\begin{array}{c} p_{\theta }\left(x_{t},h_{t},m_{t},O_{t}|z_{t},a_{t}\right)=p_{\theta }\left(x_{t}|z_{t}\right)p_{\theta }\left(h_{t}|z_{t}\right)p_{\theta }\left(m_{t}|z_{t}\right)p_{\theta }\left(O_{t}|z_{t},a_{t}{,h}_{t}\right) \end{array}$$

The variational distribution of a graph model can be written as the product of recognition terms $q\left(z_{t}|x_{t},a_{t},h_{t}{,m}_{t}\right)$ and policy terms $\pi (a_{t}|x_{t},h_{t},m_{-i,t})$:

(A3) $$\begin{array}{c} q\left(z_{t},a_{t}|x_{t},h_{t},m_{t}\right)\;=\;q\left(z_{t}|x_{t},a_{t},h_{t}{,m}_{t}\right)\;\pi \left(a_{t}|x_{t},h_{t},m_{-i,t}\right) \end{array}$$

Optimizing the variational lower bound (ELBO) can obtain the maximum marginal likelihood $\log p(x_{t},O_{t},h_{t},m_{t})$. With the posterior from variational distribution (Equation (A3)) and the likelihood from joint distribution (Equation (A1)), the the marginal likelihood can be calculated by Jensen’s inequality. The ELBO is:

(A4) $$\begin{array}{cc} \; & \log p\left(x_{t},O_{t},h_{t},m_{t}\right)\\ = & \int_{z_{t}}^{}\int_{a_{t}}^{}\log\;p\left(x_{t},O_{t},h_{t},m_{t}\right)q\left(z_{t},a_{t}|x_{t},h_{t},m_{t}\right)dz_{t}da_{t}\\ = & \int_{z_{t}}^{}\int_{a_{t}}^{}q\left(z_{t},a_{t}|x_{t},h_{t},m_{t}\right)\log\frac{p\left(x_{t},O_{t},h_{t},m_{t}\right)*p\left(z_{t},a_{t}|x_{t},h_{t},m_{t},O_{t}\right)}{p\left(z_{t},a_{t}|x_{t},h_{t},m_{t},O_{t}\right)}\;dz_{t}da_{t}\\ = & \int_{z_{t}}^{}\int_{a_{t}}^{}q\left(z_{t},a_{t}|x_{t},h_{t},m_{t}\right)\log\frac{p\left(z_{t},a_{t},x_{t},h_{t},m_{t},O_{t}\right)}{p\left(z_{t},a_{t}|x_{t},h_{t},m_{t},O_{t}\right)}\;dz_{t}da_{t}\\ = & \int_{z_{t}}^{}\int_{a_{t}}^{}q\left(z_{t},a_{t}|x_{t},h_{t},m_{t}\right)\log\frac{q\left(z_{t},a_{t}|x_{t},h_{t},m_{t}\right)}{q\left(z_{t},a_{t}|x_{t},h_{t},m_{t}\right)}\frac{p\left(z_{t},a_{t},x_{t},h_{t},m_{t},O_{t}\right)}{p\left(z_{t},a_{t}|x_{t},h_{t},m_{t},O_{t}\right)}\;dz_{t}da_{t}\\ = & \int_{z_{t}}^{}\int_{a_{t}}^{}q\left(z_{t},a_{t}|x_{t},h_{t},m_{t}\right)\log\frac{q\left(z_{t},a_{t}|x_{t},h_{t},m_{t}\right)}{p\left(z_{t},a_{t}|x_{t},h_{t},m_{t},O_{t}\right)}\frac{p\left(z_{t},a_{t},x_{t},h_{t},m_{t},O_{t}\right)}{q\left(z_{t},a_{t}|x_{t},h_{t},m_{t}\right)}\;dz_{t}da_{t}\\ = & \int_{z_{t}}^{}\int_{a_{t}}^{}q\left(z_{t},a_{t}|x_{t},h_{t},m_{t}\right)\log\frac{q\left(z_{t},a_{t}|x_{t},h_{t},m_{t}\right)}{p\left(z_{t},a_{t}|x_{t},h_{t},m_{t},O_{t}\right)}\;dz_{t}da_{t}\\ + & \int_{z_{t}}^{}\int_{a_{t}}^{}q\left(z_{t},a_{t}|x_{t},h_{t},m_{t}\right)\log\frac{p\left(z_{t},a_{t},x_{t},h_{t},m_{t},O_{t}\right)}{q\left(z_{t},a_{t}|x_{t},h_{t},m_{t}\right)}\;dz_{t}da_{t}\\ = & D_{\mathrm{kl}}\left(q\left(z_{t},a_{t}|x_{t},h_{t},m_{t}\right)\right|\left|p\left(z_{t},a_{t}|x_{t},h_{t},m_{t},O_{t}\right)\right)+\mathrm{ELBO} \end{array}$$

Since $\log p\left(x_{t},O_{t},h_{t},m_{t}\right)$ is fixed and KL-divergence is positive, we convert the problem into optimizing ELBO:

(A5) $$\begin{array}{cc} \; & \mathrm{ELBO}\\ = & \int_{z_{t}}^{}\int_{a_{t}}^{}q\left(z_{t},a_{t}|x_{t},h_{t},m_{t}\right)\log\frac{p\left(z_{t},a_{t},x_{t},h_{t},m_{t},O_{t}\right)}{q\left(z_{t},a_{t}|x_{t},h_{t},m_{t}\right)}\;dz_{t}da_{t}\\ = & \int_{z_{t}}^{}\int_{a_{t}}^{}q\left(z_{t},a_{t}|x_{t},h_{t},m_{t}\right)\log\frac{p\left(x_{t},h_{t},m_{t},O_{t}|z_{t},a_{t}\right)p\left(z_{t},a_{t}\right)}{q\left(z_{t},a_{t}|x_{t},h_{t},m_{t}\right)}\;dz_{t}da_{t}\\ = & \int_{z_{t}}^{}\int_{a_{t}}^{}q\left(z_{t},a_{t}|x_{t},h_{t},m_{t}\right)\log\frac{p\left(z_{t},a_{t}\right)}{q\left(z_{t},a_{t}|x_{t},h_{t},m_{t}\right)}\;dz_{t}da_{t}\\ + & \int_{z_{t}}^{}\int_{a_{t}}^{}q\left(z_{t},a_{t}|x_{t},h_{t},m_{t}\right)\log p\left(x_{t},h_{t},m_{t},O_{t}|z_{t},a_{t}\right)\;dz_{t}da_{t}\\ = & -D_{\mathrm{kl}}(q\left(z_{t},a_{t}|x_{t},h_{t},m_{t}\right)|\left|p\left(z_{t},a_{t}\right)\right)+E_{z_{t},a_{t}∼q\left(z_{t},a_{t}|x_{t},h_{t},m_{t}\right)}[\log p\left(x_{t},h_{t},m_{t},O_{t}|z_{t},a_{t}\right) \end{array}$$

The first term in ELBO is $-D_{\mathrm{kl}}\left(q\left(z_{t},a_{t}|x_{t},h_{t},m_{t}\right)\right|\left|p\left(z_{t},a_{t}\right)\right)$

(A6) $$\begin{array}{cc} \; & -D_{\mathrm{kl}}\left(q\left(z_{t},a_{t}|x_{t},h_{t},m_{t}\right)\right|\left|p\left(z_{t},a_{t}\right)\right)\\ = & \int_{z_{t}}^{}\int_{a_{t}}^{}q\left(z_{t},a_{t}|x_{t},h_{t},m_{t}\right)\log\frac{p\left(z_{t},a_{t}\right)}{q\left(z_{t},a_{t}|x_{t},h_{t},m_{t}\right)}\;dz_{t}da_{t}\\ = & E_{a_{t}∼\pi \left(\cdot |x_{t},h_{t},m_{-i,t}\right)}(-D_{\mathrm{kl}}(q\left(z_{t}|x_{t},a_{t},h_{t},m_{t}\right)||p\left(z_{t}\right)))\\ \; & +E_{a_{t}∼\pi \left(\cdot |x_{t},h_{t},m_{-i,t}\right)}\left[E_{z_{t}∼q\left(\cdot |x_{t},a_{t},h_{t}{,m}_{t}\right)}(\log p(a_{t})+H\left(\pi \left(a_{t}|x_{t},h_{t},m_{-i,t}\right)\right)\right] \end{array}$$

where, a prior action $\log p(a_{t})$ is assumed to be a constant term, which can be ignored when optimizing the objective.

The second term in ELBO is $E_{z_{t},a_{t}∼q\left(z_{t},a_{t}|x_{t},h_{t},m_{t}\right)}[\log p\left(x_{t},h_{t},m_{t},O_{t}|z_{t},a_{t}\right)$

(A7) $$\begin{array}{cc} \; & E_{z_{t},a_{t}∼q\left(z_{t},a_{t}|x_{t},h_{t},m_{t}\right)}[\log p\left(x_{t},h_{t},m_{t},O_{t}|z_{t},a_{t}\right)\\ = & \int_{z_{t}}^{}\int_{a_{t}}^{}q\left(z_{t},a_{t}|x_{t},h_{t},m_{t}\right)\log p\left(x_{t},h_{t},m_{t},O_{t}|z_{t},a_{t}\right)\;dz_{t}da_{t}\\ = & E_{a_{t}∼\pi \left(\cdot |x_{t},h_{t},m_{-i,t}\right)}\left[E_{z_{t}∼q\left(\cdot |x_{t},a_{t},h_{t}{,m}_{t}\right)}\left(\log p_{\theta }\left(x_{t}|z_{t}\right)\right)\right]\\ + & E_{a_{t}∼\pi \left(\cdot |x_{t},h_{t},m_{-i,t}\right)}\left[E_{z_{t}∼q\left(\cdot |x_{t},a_{t},h_{t}{,m}_{t}\right)}\left(\log p_{\theta }\left(h_{t}|z_{t}\right)\right)\right]\\ + & E_{a_{t}∼\pi \left(\cdot |x_{t},h_{t},m_{-i,t}\right)}\left[E_{z_{t}∼q\left(\cdot |x_{t},a_{t},h_{t}{,m}_{t}\right)}\left(\log p_{\theta }\left(m_{t}|z_{t}\right)\right)\right]\\ + & E_{a_{t}∼\pi \left(\cdot |x_{t},h_{t},m_{-i,t}\right)}\left[E_{z_{t}∼q\left(\cdot |x_{t},a_{t},h_{t}{,m}_{t}\right)}\left(r\left(z_{t},a_{t}{,h}_{t},m_{-i,t}\right)\right)\right] \end{array}$$

We add the results of the above two items together to obtain ELBO as follow:

(A8) $$\begin{array}{cc} \mathrm{ELBO}= & E_{a_{t}∼\pi \left(\cdot |x_{t},h_{t},m_{-i,t}\right)}\left[E_{z_{t}∼q\left(\cdot |x_{t},a_{t},h_{t}{,m}_{t}\right)}(\log p_{\theta }\left(x_{t}|z_{t}\right)+\log p_{\theta }\left(h_{t}|z_{t}\right)\right.\\ \; & \left.+\log p_{\theta }\left(m_{t}|z_{t}\right)+r\left(z_{t},a_{t}{,h}_{t},m_{-i,t}\right)-\log\pi \left(a_{t}|x_{t},h_{t},m_{-i,t}\right)\right]\\ \; & +E_{a_{t}∼\pi \left(\cdot |x_{t},h_{t},m_{-i,t}\right)}[-D_{\mathrm{kl}}\left(q\left(z_{t}|x_{t},a_{t},h_{t}\right)|\left|p\left(z_{t}\right)\right)\right] \end{array}$$

We take the entropy of the policy out separately and rewrite the ELBO formula again:

(A9) $$\begin{array}{cc} \mathrm{ELBO}= & E_{a_{t}∼\pi \left(\cdot |x_{t},h_{t},m_{-i,t}\right)}\;\left[\;E_{z_{t}∼q\left(\cdot |x_{t},a_{t},h_{t}{,m}_{t}\right)}\;\left(\;\log p_{\theta }\left(x_{t}|z_{t}\right)\;+\;\log p_{\theta }\left(\;h_{t}|z_{t}\;\right)\;+\;\log p_{\theta }\;\left(m_{t}|z_{t}\right)\;\right.\right.\\ \; & \left.\left.+r\left(z_{t},a_{t}{,h}_{t},m_{-i,t}\right)\right)\right]+E_{z_{t}∼q\left(\cdot |x_{t},a_{t},h_{t}{,m}_{t}\right)}[H\left(\pi \left(a_{t}|x_{t},h_{t},m_{-i,t}\right)\right]\\ \; & +E_{a_{t}∼\pi \left(\cdot |x_{t},h_{t},m_{-i,t}\right)}[-D_{\mathrm{kl}}\left(q\left(z_{t}|x_{t},a_{t},h_{t}\right)|\left|p\left(z_{t}\right)\right)\right] \end{array}$$

The first part of the ELBO is about latent variable *z*:

(A10) $$\begin{array}{cc} J_{M}\;\left(\theta ,\phi \right)\;=\; & E_{a_{t}∼\pi \left(\;\cdot |x_{t},h_{t},m_{-i,t}\right)}\left[\;E_{z_{t}\;∼q\left(\cdot |x_{t},a_{t},h_{t}{,m}_{t}\;\right)}\left(\log p_{\theta }\left(\;x_{t}|z_{t}\right)+\log p_{\theta }\left(\;h_{t}|z_{t}\right)+\log p_{\theta }\left(\;m_{t}|z_{t}\right)\;\right)\right]\\ \; & +E_{a_{t}∼\pi \left(\cdot |x_{t},h_{t},m_{-i,t}\right)}[-D_{\mathrm{KL}}\left(q_{\phi }\left(z_{t}|x_{t},a_{t},h_{t}\right)|\left|p_{\theta }\left(z_{t}\right)\right)\right] \end{array}$$

The other part equation is maximum entropy reinforcement learning:

(A11) $$\begin{array}{c} J_{\pi }\left(\varphi \right)=E_{a_{t}∼\pi \left(\;\cdot |x_{t},h_{t},m_{-i,t}\right)}\left[E_{z_{t}∼q\left(\cdot \;|\;x_{t},a_{t},h_{t}{,m}_{t}\right)}\left(\;r\left(\;z_{t},a_{t}{,h}_{t},m_{-i,t}\right)+H\left(\pi \left(\;a_{t}|x_{t},h_{t},m_{-i,t}\;\right)\right)\right)\right] \end{array}$$

## Appendix B. Implementation and Experimental Details

The environments we used in our experiments for StarCraft are shown in Figure 5, and the two environments we designed are adapted from SMAC [63]. The agent’s task is to navigate to a randomly initialized goal in maps. Figure 5a is a maze game where we set up grooves on the map. These grooves are connected to form the paths that the agent walks. The target positions are set randomly on these paths. Figure 5b is a tree search navigation task. The navigation target will randomly appear on the leaf nodes. We designed two scenes of different difficulties on the tree map. One is a tree structure with a depth of 3, and the other is 4, which is a little more complicated.

In all maps, we design two allies and one enemy. Two allied teammates have their roles: a navigation agent that interacts with the environment and the other is an oracle agent that provides target information and relative positions. The enemy agent is designed as an immobile navigation target.

For the observation setting, the oracle agent’s observations: the position of the target, the position of the navigation agent, the terrain information around the target, and whether it is an accessible road. The observation of the navigation agent is only locally observed terrain information and its position information.

In setting the reward value, our reward value function is designed as:$r_{t}=1_{[\text{reached-goal}]}\cdot r_{\mathrm{goal}}+r_{\mathrm{closer}}+r_{\text{time-penalty}}+1_{\left[\text{send-message}\right]}\cdot r_{\text{comm-penalty}}$. In the setting, the agent’s reward for navigating to a goal is $r_{\mathrm{goal}}=1$, and the reward for close distance is $r_{\mathrm{closer}}=\Delta \cdot 0.005$, where $\Delta =-({\mathrm{dis}}_{t}-{\mathrm{dis}}_{t-1})$ and ${\mathrm{dis}}_{t}$ is the distance from the agent to the target point at time *t*. Our single-step penalty for the agent is 0.1, $r_{\text{time-penalty}}=-0.1$. In default settings, the penalty for communication is 0.5, $r_{\text{comm-penalty}}=-0.5$. In the selection of parameters we used the parameters in Table A1.

**Table A1:** Parameter setting in StarCraft environment.

Parameters	Value
Critic learning rate	0.005
Actor learning rate	0.005
Batch size	32
Discount factor	0.99
VEA network learning rate	0.0001

In the Habitat environment, we use the same hyperparameter settings as in the CoMon [19], where we set the communication penalty term in the reward value to $r_{\text{comm-penalty}}=-0.01$. The learning rate set in the variational network is 0.0001.

We designed the following agent models for Habitat:

- NoCom (i.e., “No communication”): It means the performance of the navigation agent without relying on the oracle agent, which can be used as a model without communication.
- U-Comm (i.e., “Unstructured communication”): the agent delivers real-valued vector messages. Oracle’s information is directly encoded by the linear layer to form information and send it to the navigation agent.
- S-Comm (i.e., “Structured communication”): the agent has a vocabulary consisting of *K* words, implemented as a learnable embedding. These probabilities are obtained by passing information to the linear and softmax layers. On the receiving end, the agent decodes these incoming message probabilities by linearly combining its word embeddings using the probabilities as weights.
- S-Comm&CIC (i.e., “Structured communication with Causal Inference model”): Combining our causal inference network on the basis of U-Comm.
- U-Comm&CIC (i.e., “Unstructured communication with Causal Inference model”): Combining our causal inference network on the basis of S-Comm.

We designed the following agent models for Starcraft environment:

- SACwithoutComm: model without communication.
- SACwithComm, only using SAC algorithm with the communication.
- SACwithLatentAndComm: adding latent variable model and communication using VAE to SAC algorithm.
- CICM (our method): leveraging causal inference and VAE model.
